# Evaluation of a new Q-switched Nd:YAG laser on premacular hemorrhage

**DOI:** 10.1186/s12886-023-02876-7

**Published:** 2023-04-07

**Authors:** Zhaoan Su, Luyao Tong, Jingliang He, Lijuan Wang, Jun Liu, Xiaoyun Fang, Li Zhang

**Affiliations:** 1 Eye Center of the Second Affiliated Hospital, School of Medicine, Zhejiang University, Hangzhou, Zhejiang 310009 China; 2 Department of Ophthalmology, The First Affiliated Hospital of Ningbo University, Ningbo, Zhejiang 315000 China

**Keywords:** Premacular hemorrhage, Nd:YAG laser, Q-switched laser, Case series

## Abstract

**Background:**

Premacular hemorrhage is among the causes of sudden deterioration of visual acuity. This study aimed to investigate the therapeutic outcomes of a new Q-switched Nd:YAG laser on premacular hemorrhage.

**Methods:**

Retrospective, case series study of 16 eyes from 16 patients diagnosed with premacular hemorrhage, including 3 cases of Valsalva retinopathy, 8 cases of retinal macroaneurysm, 3 cases of diabetic retinopathy, 1 case of trauma-related hemorrhage and 1 case with leukemia. A 1064nm Q-switched Nd:YAG laser was performed to puncture the posterior hyaloid and inner limiting membrane to drain the hemorrhage.

**Results:**

The success rate of 16 patients with premacular hemorrhage drainage was 100% in this study. Improved visual acuity was observed in each patient.

**Conclusions:**

In this case series of 16 patients, the new Q-switched Nd:YAG laser was successful in draining premacular hemorrhage with no serious complications.

## Background

Premacular hemorrhage is the accumulation of hemorrhage in the vitreo-retinal interface, located in front of the macular [1–3]. It generally occurs in diabetic retinopathy [4, 5], ruptured retinal macroaneurysm [6–9], Terson’s syndrome[10], or ruptured retinal vessels after distant trauma or physical sport (Valsalva) [11, 12]. Premacular hemorrhage in small amounts may be managed conservatively [12]. Dense premacular hemorrhage or combined with proliferation usually needs surgical intervention [13–15]. The use of Nd:YAG laser has been reported in the drainage of premacular hemorrhage by puncturing the anterior surface of the hemorrhagic cyst [16–18].

The Q-switched Nd:YAG laser produces near-infrared radiation at 1064 nm, with each pulse between 2 and 14 nanoseconds [19]. It is clinically used to disintegrate hemorrhagic cysts by ionization, which substitutes surgical interventions. Compared with mode-locked Nd:YAG laser, Q-switched Nd:YAG laser has the advantages of lower focused spot irradiance and less damage to the retina [20]. Treatment of premacular hemorrhage with Q-switched Nd:YAG laser has been reported in several studies [5, 16]. Despite the above advantages, traditional Q-Switched Nd:YAG has a risk of chorioretinal injury when the focus is close to the retina (1.5-3 mm) [21]. The appearance of non-clearing vitreous hemorrhage and progress into retinal detachment were uncommon but require further vitrectomy [22]. A complication with macular hole was reported in a long-term follow-up study [23]. Ultra Q reflex (Ellex Medical Pty Ltd, Australia), a new Q-switched Nd:YAG laser, has been applied in the treatment of floaters in the posterior vitreous in recent years. Its laser cavity was capable of producing a 4 nanosecond Ultra Gaussian pulse at a high peak energy and achieves optical breakdown at a low energy level. Investigating its therapeutic application on premacular hemorrhage could be valuable.

The purpose of this study was to evaluate the therapeutic effect of this new Q-switched Nd:YAG laser on premacular hemorrhage. We included premacular hemorrhage cases with diabetic retinopathy, ruptured retinal macroaneurysm, Valsalva retinopathy and et al in this study.

## Methods

This study has been approved by the Institutional Review Board of the Second Affiliated Hospital, School of Medicine, Zhejiang University in Hangzhou, China. All patients provided written informed consent.

This retrospective, case series study enrolled premacular hemorrhage patients between May 2017 and January 2023 at the Eye Center of the Second Affiliated Hospital, School of Medicine, Zhejiang University. The inclusion criteria include (1) hemorrhage accumulated in front of macular, diagnosed as premacular hemorrhage; (2) the new Q-switched Nd:YAG laser (Ultra Q Reflex, Ellex Medical Pty Ltd, Australia) was applied for hemorrhage drainage within one week. Patients with a history of ocular surgery or infection were excluded.

Premacular hemorrhage was diagnosed based on optical coherence tomography (OCT) (Stratus OCT, Carl Zeiss Meditec, USA; Spectralis OCT, Heidelberg, Germany), fundus photographs (Daytona, Optos PLC, Dunfermline, United Kingdom), fluorescein angiography (FFA) (Heidelberg Engineering, Heidelberg, Germany) and posterior segment examination by experienced ophthalmologists. Best-corrected visual acuity (BCVA) was examined during follow-up and converted to logMAR equivalent [24, 25]. The new 1064 nm Q-switched Nd:YAG laser was performed to create an opening at the highest anterior surface of the hemorrhagic cyst, while avoiding damage to macular or adjacent vessels. Laser exposures started with an energy of 3 mJ and gradually increases until successful drainage. Several shots were tried per energy stage by the same experienced ophthalmologist. Once a successful puncture was achieved, the patient was asked to remain in a sitting position for two hours to drain the hemorrhage by gravity. All patients were asked to follow up within 1 week. Successful draining was the primary outcome of this study, considered as the disappearance of hemorrhage from the fovea area at 1-week follow-up [6].

Statistical analyses were performed with SPSS version 26.0 (SPSS, Inc., Chicago, IL, USA). The pre-and post-laser BCVA values were converted to logMAR equivalent for statistical analysis and compared by compared t-test. P < 0.05 was considered statistically significant.

## Results

Sixteen eyes from 16 patients (6 males and 10 females) of premacular hemorrhage were enrolled in this study (Table 1). Three of them were diagnosed with diabetic retinopathy, 8 with retinal macroaneurysm, 1 with trauma-related hemorrhage, 3 with Valsalva retinopathy, and 1 with leukemia. The follow-up period ranged from 1 week to 2 years (median 2 months). The mean size of the hemorrhage pre-treatment was 3.81 disc diameters, which was measured based on fundus photographs. The BCVA ranged from counting fingers at 0.5 m to counting fingers at 1 m.

**Table 1:** Summary of patients’ characteristics

Case	Age (year)	Diagnosis	Eye	Size (DD)	BCVA		Number of laser shots*	Successful puncture energy (mJ)		Follow-up
					pre-laser	post-laser		per shot	total	
1	67	Retinal macroaneurysm	OS	3	CF/1m	20/80	3	3.8	11.4	1 week
2	62	Retinal macroaneurysm	OD	2	CF/1m	20/63	2	3.6	7.2	2 months
3	71	Retinal macroaneurysm	OD	3	CF/1m	20/100	3	3.4	10.2	1 month
4	67	Retinal macroaneurysm	OS	5	CF/0.5 m	20/40	4	3.6	14.4	10 months
5	23	Retinal macroaneurysm	OS	5	CF/0.5 m	20/32	2	3.4	6.8	2 months
6	63	Retinal macroaneurysm	OS	3	CF/1m	20/100	2	3.0	6.0	3 weeks
7	68	Retinal macroaneurysm	OS	4	CF/1m	20/40	2	2.6	5.2	2 months
8	64	Retinal macroaneurysm	OD	4	CF/0.5 m	20/40	1	2.8	2.8	2 months
9	53	Diabetic retinopathy	OD	3	CF/0.5 m	20/63	5	3.6	18.0	3 months
10	61	Diabetic retinopathy	OD	5	CF/1m	20/100	6	3.8	22.8	2 weeks
11	43	Diabetic retinopathy	OD	6	CF/0.5 m	20/100	2	3.2	6.4	2 months
12	36	Valsalva retinopathy	OD	4	CF/0.5 m	20/25	1	3.0	3.0	6 months
13	28	Valsalva retinopathy	OD	4	CF/1m	20/20	2	3.0	6.0	2 years
14	48	Valsalva retinopathy	OD	4	CF/1m	20/25	1	3.2	3.2	1 week
15	33	Trauma-related hemorrhage	OD	3	CF/1m	20/40	3	3.2	9.6	1 week
16	32	Leukemia	OS	3	CF/1m	20/25	1	2.6	2.6	1 month

DD = disc diameter; OS = left eye; OD = right eye; BCVA = best-corrected visual acuity; CF = count fingers

* Number of laser shots represents the total number of the laser shots at the energy level when puncture was achieved

All patients underwent 1 to 6 (mean 2.50) laser shots, with the energy ranging from 3.0 to 3.8 mJ (mean 3.39 mJ per shot). The hemorrhage drainage rate was 100% in 16 patients. The mean BCVA was significantly improved at 1 week and remained stable during follow-up (range from 20/100 to 20/20). The characteristics of the patients were summarized in Table 1. Statistical analysis of BCVA before and 1 week after the treatment was presented in Table 2.

**Table 2:** The statistical analysis of visual acuity before and 1 week after treatment

Case	Age (year)	BCVA logMAR equivalent	
		pre-laser	post-laser
1	67	1.70	0.60
2	62	1.70	0.50
3	71	1.70	0.70
4	67	1.85	0.30
5	23	1.85	0.20
6	63	1.70	0.70
7	68	1.70	0.30
8	64	1.85	0.30
9	53	1.85	0.50
10	61	1.70	0.70
11	43	1.85	0.70
12	36	1.85	0.10
13	28	1.70	0.00
14	48	1.70	0.10
15	33	1.70	0.30
16	32	1.70	0.10
Mean BCVA		1.756 ± 0.075	0.381 ± 0.248*

BCVA = best-corrected visual acuity

* p < 0.001

Fourteen of 16 patients had residual hemorrhage at the lower edge of the hemorrhagic cyst for a short term after laser. Peripheral hemorrhage was found in Case 9 of diabetic retinopathy. Vitreo-retinal traction exacerbated along with the spontaneous absorption of the hemorrhage. Partial subretinal hemorrhage was observed in Case 4 of retinal macroaneurysm at 4 months follow-up. Hard intraretinal exudation was observed at 10 months in this patient. Complications, including macular hole, retinal detachment, or hemorrhage in the retina or choroid, were not found in this study. None of these patients underwent additional surgical intervention during the follow-up.

### Case presentation

**Fig. 1:**
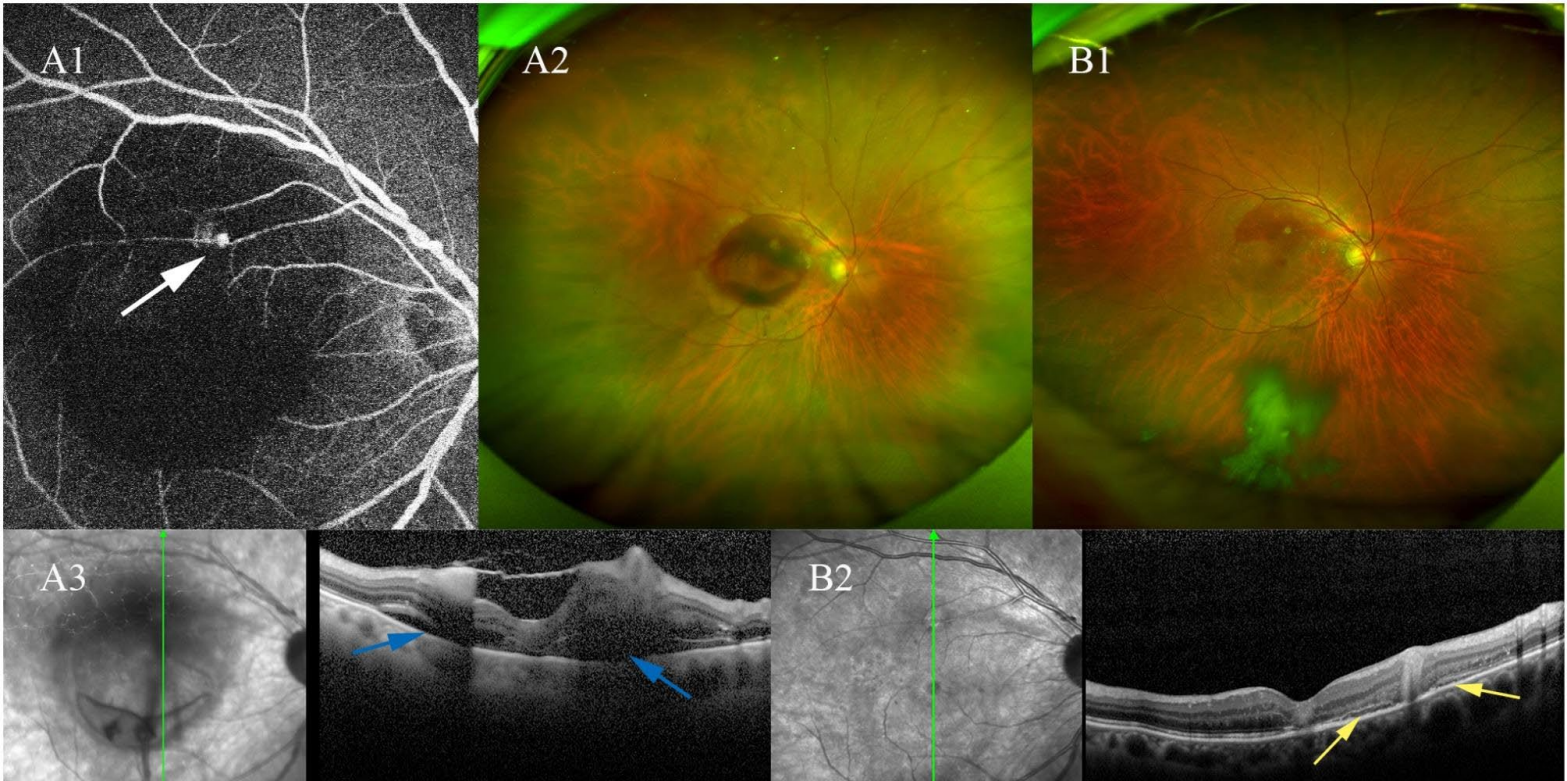
Case 3: a female with ruptured retinal macroaneurysm. (A): FFA (A1), fundus photographs (A2), and vertical scanning OCT pre-laser (A3). In the arterial stage, blocked fluorescence was observed due to the premacular hemorrhage. The retinal macroaneurysm showed a focal nodular hyperfluorescence (white arrow). Subretinal fluid was observed (blue arrow). (B): fundus photographs (B1) and OCT (B2) 1 month after treatment. High reflection was observed at the site of the macroaneurysm (yellow arrow)

Case 3 was a 71-year-old female diagnosed with retinal macroaneurysm by FFA. OCT scanning revealed pre-retinal hemorrhage and subretinal fluid. After the drainage of premacular hemorrhage with the new Q-switch Nd:YAG laser, we also performed 523 nm laser photocoagulation to the macroaneurysm. Disruption of the neuroretina structure of the fovea was observed 1 week after treatment (Fig. 1).

**Fig. 2:**
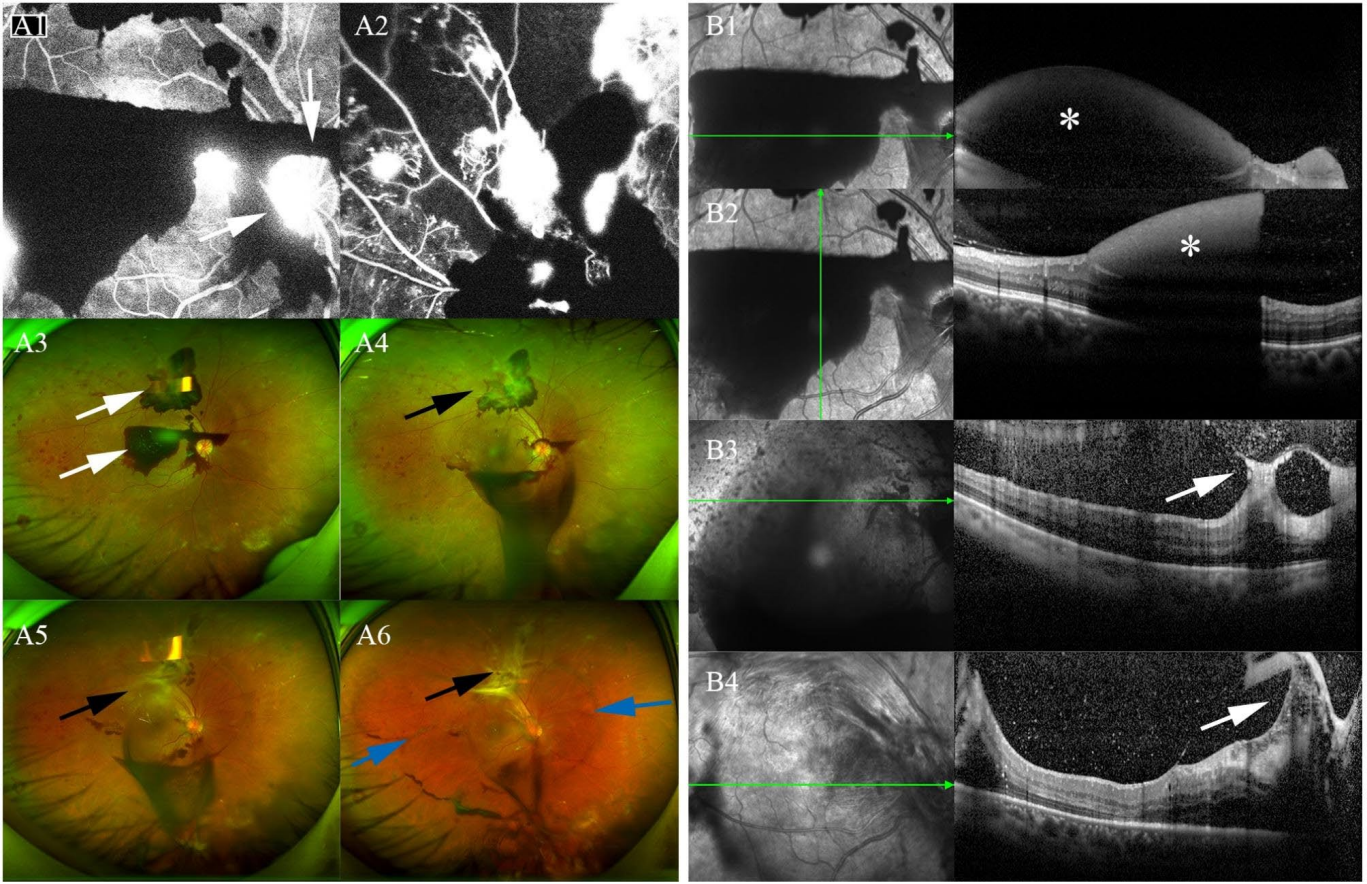
Case 9: a 53-year-old male with diabetic retinopathy. Examinations were performed 1 day before (A1, A2, A3, B1, and B2), 1 week after (A4 and B3), 1 (A5) and 2 months (A6 and B4) after the treatment. The optic disc was around by dense hemorrhage (white arrow) (A1, A3). FFA showed no perfusion area and neovascularization (A2) The proliferation got severe secondary to residual preretinal hemorrhage (black arrow) (A4, A5, and A6). The detached posterior hyaloid presented as an elliptic boundary (blue arrow). The dense preretinal hemorrhage was shown in horizontal and vertical scan lines (star) (B1 and B2). The fibrosis proliferation got serious during follow-up (B3 and B4)

Case 9 was a 53-year-old male with diabetic retinopathy. Fundus photographs revealed an accumulation of hemorrhage in the macular area and around the optic disc. Hemorrhage beneath ILM was confirmed by OCT scanning. After successful drainage of premacular hemorrhage, the development of proliferation close to the superior temporal branch artery was noted during the follow-up at 2 months (Fig. 2).

**Fig. 3:**
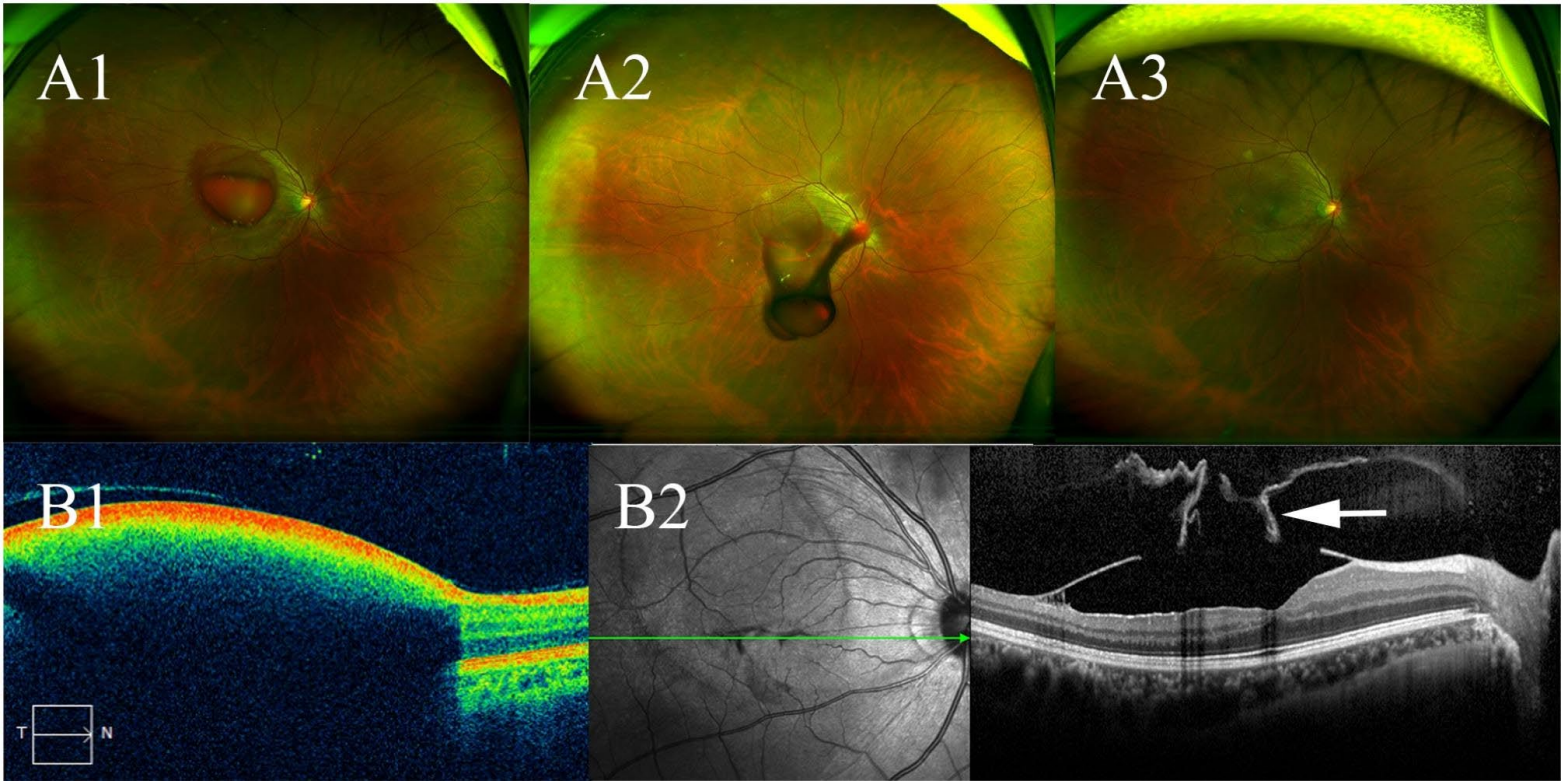
Case 13: a case with Valsalva retinopathy. Examinations were performed 1 day before (A1 and B1), the day of the treatment (A2), and 2 years follow-up (A3 and B2). A vitreous separation and rupture of the ILM were observed (B2, white arrow)

Case 13 was a 28-year-old female diagnosed with Valsalva retinopathy. A vitreous separation with a sizable hole in the ILM was observed with only 2 shots of the new Q-switch Nd:YAG laser (Fig. 3).

**Fig. 4:**
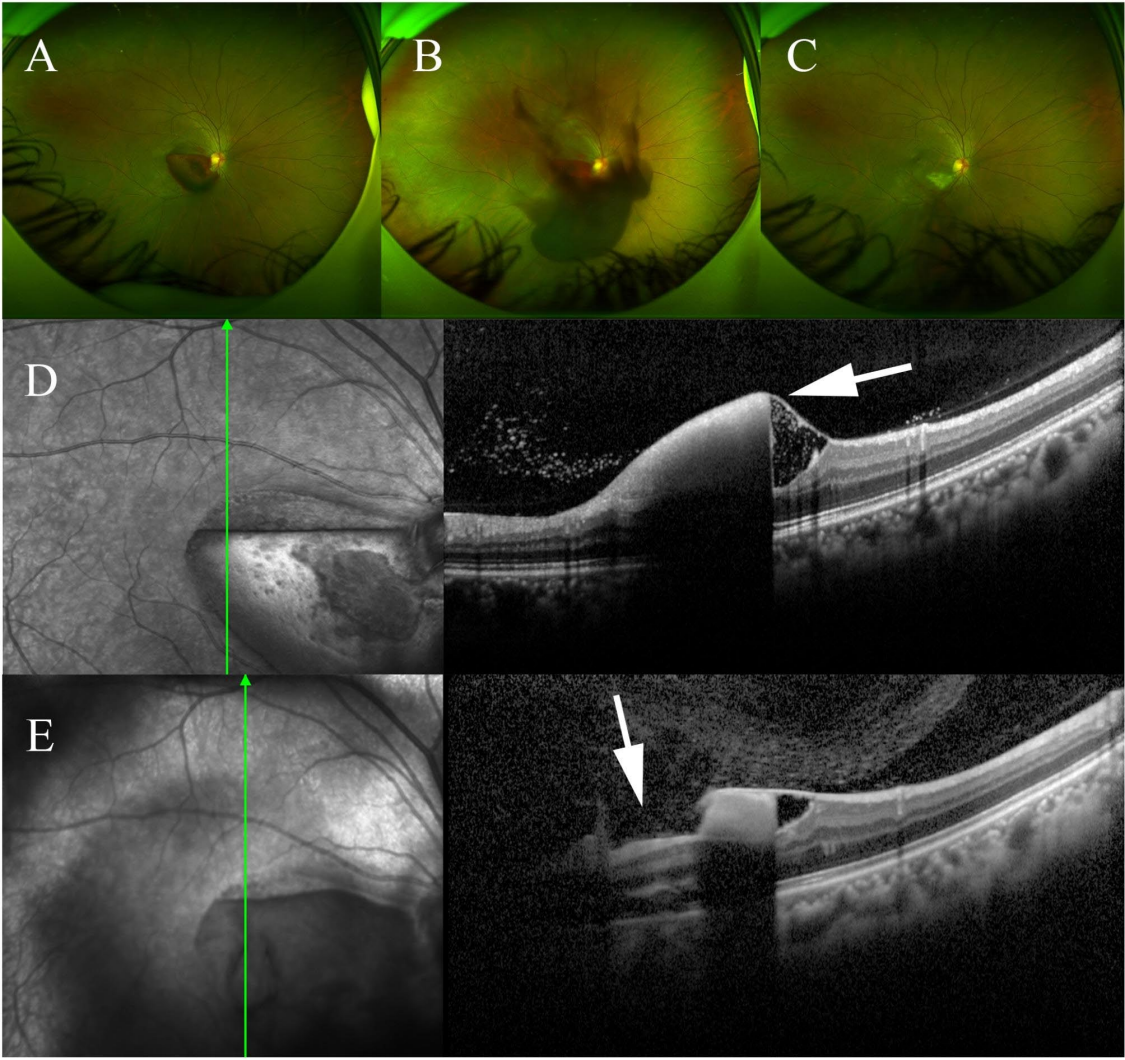
Case 15: a case with trauma-related hemorrhage. Examinations were performed 1 day before (A and D), the day of the treatment (B and E), and 1 week after the treatment (C). The boundary between thin and dense hemorrhage due to gravity was observed (D, white arrow). The puncture opening in the ILM and the normal retinal structure beneath (E, white arrow)

Case 15 was a 33-year-old female diagnosed with trauma-related premacular hemorrhage. No serious complications were observed after the treatment during the follow-up (Fig. 4).

## Discussion

Previous studies of preretinal hemorrhage treated with traditional Nd:YAG have demonstrated varying effective rates in hemorrhage drainage and incidence of complications. Our study found that the new Q-switched Nd:YAG laser has a high rate of premacular hemorrhage drainage without energy-related retinal or choroidal damage.

Premacular hemorrhage usually presents as a cyst of hemorrhage in front of the macular, resulting in impairs of central vision. The rupture of retinal macroaneurysm is the primary cause of premacular hemorrhage, usually presenting as dense blood accumulation [8, 26, 27]. Visual acuity may be worse when combined with intraretinal and subretinal hemorrhage, or macular edema and exudation [6, 7, 26, 28]. This study enrolled 8 cases with ruptured retinal macroaneurysm. Drainage of the premacular hemorrhage was successful in all patients. However, there was one case with subretinal hemorrhage in SD-OCT, suggesting a poor visual outcome. Premacular hemorrhage caused by Valsalva retinopathy is usually accompanied by a history of elevated vascular pressure [11], which usually results in a better prognosis and fewer complications than patients with other causes [18, 22, 23]. Patients with Valsalva retinopathy and trauma-related hemorrhage in our study did not have noticeable complications after treatment. In proliferative diabetic retinopathy, the premacular hemorrhage usually presents dense and is accompanied by vitreous hemorrhage [16]. Retinal neovascularization, macular edema, extensive rebleeding, and even retinal detachment are the primary causes of loss of visual acuity due to the pathology of proliferative diabetic retinopathy, which necessitates vitrectomy or intraocular injection of anti-vascular endothelial growth factor [16, 29]. Even if the premacular hemorrhage is successfully drained, the visual acuity is partial. The structure of the fovea and the function of photoreceptors are key factors in determining vision [30]. After successful drainage, the macular structure could be examined by OCT scanning. The necessity of surgical intervention can be evaluated according to the follow-up outcomes and may help to avoid unnecessary procedures. There are few reports of pre-macular hemorrhage in leukemia patients [27]. We report a premacular hemorrhage case with leukemia, who obtained a satisfactory result after treatment.

Previous studies have investigated the effectiveness of conservative observation, membranotomy, and vitrectomy for premacular hemorrhage [12–15]. Instantly removing the hemorrhage and potential epiretinal membranes by vitrectomy may lead to tractional retinal detachment. However, it might increase the risk of infection due to the incision of the eyeball, destroy the natural support and barrier functions of the vitreous body, and increase the risk of complications by postoperative glucocorticoid usage. Compared with vitrectomy, locked and traditional Q-switched Nd:YAG laser has the advantage of preserving the integrity of the eyeball which avoids possible surgical complications, shortens the treatment process, and lowers the dose of glucocorticoids [18, 22]. However, their optical design makes it impossible to accurately target the laser burst point close to the retina. Rebleeding, which implies damage to the retina or choroid, was reported in cases of diabetic retinopathy, Valsalva retinopathy, and Eales’ disease after laser [14, 22, 23, 31]. An epiretinal membrane may also occur after laser irradiation on the posterior hyaloid [32]. In this study, a new Q-switched Nd:YAG laser, with continuously variable laser energy and optimized visualization, achieves low processing energy and ensures accurate laser focus. With this laser instrument, chorioretinal injury and rebleeding were prevented during the procedure of puncturing the hemorrhagic cyst at its highest anterior surface. Therefore, this new Q-switched Nd:YAG laser can be a safe therapeutic option, except in those cases with severe retinal traction, which usually requires surgical intervention.

Unsuccessful hemorrhage drainage and non-clearing vitreous hemorrhage are the main factors affecting the success rate of Nd:YAG laser treatment for premacular hemorrhage. In our study, there were 40 laser points in 16 patients with an average energy of 3.39 mJ, which ensured a 100% drainage rate with the energy far lower than in previous reports [22, 23]. Dense preretinal hemorrhage was observed with slower drainage by the procedure of puncturing the hemorrhagic cyst at its highest anterior surface fundus photographs and OCT. Hemorrhage draining into the vitreous was even visible 1 month after treatment (Fig. 2). We speculate that the success rate might relate to the density of hemorrhage. Undoubtedly, larger puncture size contributed to successful drainage. In this study, OCT scanning revealed that the puncture opening’s width was usually 1–2 times the thickness of the retina (Fig. 3). More laser spots and higher laser energy are required for patients with a thicker membrane of the anterior cyst surface. Increased density of the vitreous cortex, aggregated collagen fibers, and morphological changes of hyalocytes were usually observed in diabetic retinopathy patients [33]. Fibrovascular neovascularization on the retinal surface and adhered to ILM was also observed in diabetic retinopathy with preretinal hemorrhage [34]. The diabetic retinopathy cases involved in this study (cases 9 and 10) exhibited a similar diameter of the premacular hemorrhage as other cases but required the maximum amount of laser energy to achieve successful drainage. The successful puncture energies of these two patients are 3.6 and 3.8 mJ respectively, which effectively prevented tissue damage caused by high laser energy.

There are several limitations to this study. The follow-up period was relatively short and the long-term complications need further observation. We only observed cases of premacular hemorrhage in retinal microaneurysm, Valsalva retinopathy, diabetic retinopathy, trauma-related, and leukemia cases. The therapeutic effect on premacular hemorrhage with other causes needs to be further explored. In addition, a prospective study with long-term follow-up would better investigate the effectiveness and safety of this new Q-switched Nd-YAG laser in the treatment of premacular hemorrhage in the future.

## Conclusions

In this case series of 16 patients, the new Q-switched Nd:YAG laser was successful in draining premacular hemorrhage with no serious complications.

## Data Availability

The datasets used and analyzed during the current study are available from the corresponding author upon reasonable request.

## Abbreviations

ILM: Internal limiting membrane
OCT: Optical coherence tomography
BCVA: Best-corrected visual acuity
FFA: fluorescein angiography
